# 不同病理亚型浸润性非黏液腺癌的研究进展

**DOI:** 10.3779/j.issn.1009-3419.2022.102.51

**Published:** 2023-01-20

**Authors:** Ruke TANG, Lina BI, Bingquan XIANG, Lianhua YE, Ying CHEN, Guangjian LI, Guangqiang ZHAO, Yunchao HUANG

**Affiliations:** ^1^650118 昆明，昆明医科大学第三附属医院胸外一科; ^1^Department of Thoracic Surgery I, Third Affiliated Hospital of Kunming Medical University, Kunming 650118, China; ^2^650032 昆明，昆明医科大学第一附属医院肾脏内科; ^2^Department of Nephrology, First Affiliated Hospital of Kunming Medical University, Kunming 650032, China; ^3^650118 昆明，昆明医科大学第三附属医院重症医学科; ^3^Department of Intensive Care Unit, Third Affiliated Hospital of Kunming Medical University, Kunming 650118, China

**Keywords:** 肺肿瘤, 浸润性腺癌, 浸润性非黏液腺癌, 病理亚型, Lung neoplasms, Invasive adenocarcinoma, Invasive non-mucinous adenocarcinoma, Pathological subtypes

## Abstract

肺癌是当今世界癌症死亡的首要原因，腺癌是肺癌中最常见的组织病理学类型。2021年5月世界卫生组织（World Health Organization, WHO）发布第5版《WHO胸部肿瘤分类》，根据组织学特点将浸润性非黏液腺癌（invasive non-mucinous adenocarcinoma, INMA）分为贴壁生长型腺癌、腺泡型腺癌、乳头型腺癌、实体型腺癌和微乳头型腺癌。这5种病理亚型在临床特征、治疗及预后等方面均有所差异，充分了解不同病理亚型的特点对肺腺癌患者的临床诊断、治疗方案的选择及疾病复发、进展等预后情况的判断具有至关重要的作用。本文将就不同病理亚型的INMA的分级系统、形态学、影像学预测、淋巴结转移、手术、化疗、靶向及免疫治疗做一综述。

## Abstract (en)

Lung cancer is the leading cause of cancer death in the world today, and adenocarcinoma is the most common histopathological type of lung cancer. In May 2021, World Health Organization (WHO) released the 5^th^ edition of the WHO classification of thoracic tumors, which classifies invasive non-mucinous adenocarcinoma (INMA) into lepidic adenocarcinoma, acinar adenocarcinoma, papillary adenocarcinoma, solid adenocarcinoma, and micropapillary adenocarcinoma based on its histological characteristics. These five pathological subtypes differ in clinical features, treatment and prognosis. A complete understanding of the characteristics of these subtypes is essential for the clinical diagnosis, treatment options, and prognosis predictions of patients with lung adenocarcinoma, including recurrence and progression. This article will review the grading system, morphology, imaging prediction, lymph node metastasis, surgery, chemotherapy, targeted therapy and immunotherapy of different pathological subtypes of INMA.

肺癌是当今世界癌症死亡的首要原因，2020年全球估计有180万人因肺癌死亡^[1]^。同时肺癌也是我国发病率最高的恶性肿瘤，据国家癌症中心统计，2016年新发肺癌病例约54.98万例，占所有新发癌症病例的24.6%^[2]^。其中，腺癌是肺癌中最常见的组织病理学类型。2011年，国际肺癌研究协会（International Association for the Study of Lung Cancer, IASLC）、美国胸科学会（American Thoracic Society, ATS）及欧洲呼吸学会（European Respiratory Society, ERS）共同发布了肺腺癌的国际多学科分类方法。该分类方法将肺腺癌的组织病理学类型分为浸润前病变、原位腺癌、微浸润性腺癌、浸润性腺癌（invasive adenocarcinoma, IA）和浸润性腺癌变异型，并提出IA应根据主要类型分为：贴壁生长型腺癌（lepidic predominant adenocarcinoma, LPA）、腺泡型腺癌（acinar predominant adenocarcinoma, APA）、乳头状腺癌（papillary predominant adenocarcinoma, PPA）、实体型腺癌（solid predominant adenocarcinoma, SPA）和微乳头状腺癌（micropapillary predominant adenocarcinoma, MPA）五类^[3]^。2021年5月，第5版《世界卫生组织（World Health Organization, WHO）胸部肿瘤分类》（2021版分类）不再将浸润性黏液性腺癌（invasive mucinous adenocarcinoma, IMA）视为浸润性腺癌变异型的病理亚型，而是作为肺腺癌的一种组织病理学类型，并将原分类中的IA重新命名为浸润性非黏液性腺癌（invasive non-mucinous adenocarcinoma, INMA），INMA仍包括LPA、APA、PPA、SPA、MPA五类^[4,5]^。到目前为止，多项研究^[6⇓⇓⇓-10]^显示出不同病理亚型的INMA在生物学特性、临床治疗及预后等方面均有所差异，本文将从浸润性肺腺癌分级系统、形态学、影像学预测、淋巴结转移、手术、化疗、靶向及免疫治疗等方面，针对各INMA病理亚型的研究进展做一综述。

## 1 浸润性肺腺癌分级系统

因上述IA及INMA病理亚型常混合存在，2011年肺腺癌IASLC/ATS/ERS国际多学科分类^[3]^要求将IA的病理学亚型按照各亚型所占比例，以5%为增量从高到低依次列出，其中实体型及微乳头型腺癌未达5%也列出。不同病理亚型的INMA预后不同并可以预测肿瘤生物学行为。大量研究^[11⇓-13]^显示LPA的预后最佳，MPA和SPA预后最差。国外有学者^[14]^认为MPA/SPA的中位体积倍增时间为232.7天，中位质量倍增时间为221.8天。它们的肿瘤倍增时间短于其他腺癌病理亚型，与鳞状细胞癌相近，而LPA的中位体积倍增时间为1,140.6天，中位质量倍增时间为970.1天，肿瘤倍增时间长于其他亚型。MPA/SPA的快速生长可能是其预后不良的原因之一。2015年WHO^[15]^使用亚型所占比例将腺癌分为三个预后级别：低级别模式（LPA）、中级别模式（PPA或APA）、高级别模式（SPA或PPA）。复杂腺样型属于腺泡型中的一种类型，包括筛状腺、融合腺和松散腺型^[15,16]^。既往研究^[17,18]^表明复杂腺样型与较差的预后相关，故2015年WHO分类将其预后级别归为高级别模式。

但2015年WHO分类仅考虑了肺腺癌患者预后与主要组织病理学类型的关系，并未考虑到肺腺癌组织中预后级别最差的组织学类型亦与患者预后相关。因此2020年IASLC开展了一项多中心研究，该研究基于主要的组织学类型和预后最差的组织学类型的组合，建立了浸润性肺腺癌的组织学分级系统。新的IASLC分级系统分成3级：1级为高分化：贴壁生长型为主且高级别模式（实体、微乳头状或复杂腺型）不超过20%；2级为中分化：腺泡或乳头状为主且高级别模式不超过20%；3级为低分化：具有20%或以上的高级别模式。与单纯基于组织学模式的患者预后预测相比，这种新的IASLC分级系统改善了IA早期患者的预后分层^[19]^。Rokutan-Kurata等^[20]^在一个大型日本队列研究中评估了IASLC分级系统的预后价值，证明了浸润性肺腺癌的IASLC系统具有预后意义，认为IASLC分级系统将为临床医生提供更好的术后治疗信息。Deng等^[21]^进行了一个大型的、详细的亚洲人群队列研究，研究认为新的IASLC分级系统对IA的预后区分优于单纯基于组织学模式。其他研究^[22⇓-24]^也证实了新提出的IASLC分级系统在预测患者预后方面是有用的，在检测高级别恶性肿瘤方面优于旧的分级系统。2021年WHO第5版胸部肿瘤分类虽继续以5%为增量描述INMA的不同病理亚型，但不再描述为以某亚型为主的腺癌，并推荐使用IASLC分级系统^[4,5]^。

## 2 各病理亚型的形态学特征

2011年肺腺癌IASLC/ATS/ERS国际多学科分类定义了不同病理亚型IA的形态学特征^[3]^。LPA是指在一个视野下存在>0.5 cm浸润性病灶的IA。如在肿瘤中有多灶性浸润性病灶，可采用浸润性病灶的百分比之和乘以肿瘤的最大径，如数值>0.5 cm即可做出诊断，此项诊断仅用于IA^[5]^。肿瘤大小是第8版肿瘤原发灶-淋巴结-转移（tumor-node-metastasis, TNM）分期的关键因素之一^[25]^。既往研究^[26,27]^表明，对于IA，肿瘤侵袭性成分长径预测预后的效能好于肿瘤长径。2017年第8版TNM分期将肿瘤侵袭区域纳入T分期计算，伴有贴壁生长亚型的IA的肿瘤大小的测量依据侵袭灶大小而非肿瘤大小分期^[28]^。

APA是指腺泡腔及肿瘤细胞内可有黏液，但无柱状黏液细胞贴壁生长的成分。WHO虽将复杂腺体类型归于腺泡型^[5]^，但IASLC分级系统考虑到复杂腺体类型与传统腺泡类型预后不同，将其分开分级。

PPA是指为带有纤维中心的乳头状结构，WHO第5版胸部肿瘤分类认为假乳头结构形成与肺泡壁不同切面及肺泡壁的塌陷有关，手术可能是假乳头结构形成的一种原因^[5]^。

SPA是指肿瘤细胞以实性巢状或片状排列为主，注意与非角化型鳞癌和大细胞癌鉴别。部分细胞可含有细胞内黏液，黏液染色显示含有细胞内黏液肿瘤细胞数>2个/5个高倍视野。

MPA是指一种充满丝状、簇状或细长无纤维血管轴心乳头的肺泡或腺样结构，常有血管、淋巴管和间质侵犯，并可见砂粒体。近年来，有研究^[29,30]^报道一种新的丝状微乳头生长模式，呈现为纤细、蕾丝样，肿瘤内缺乏纤维血管轴心，3个以上瘤细胞高度的狭长肿瘤细胞被堆积。2021年WHO将其纳入第5版胸部肿瘤分类，并认为当计算百分比时，围绕腺管、乳头、贴壁形态的区域应计入微乳头，不计入其他亚型^[5]^。

## 3 基于影像学的病理亚型预测

不同病理亚型INMA的侵袭性以及预后不同^[31]^，早期诊断尤为重要。目前临床实践中，肺癌病理类型及亚型的鉴别主要采用侵入性活检和术后病理两种有创诊断方法^[32]^，但近年出现了大量使用影像学方法进行病理亚型预测的研究。计算机断层扫描（computed tomography, CT）是最常用的肺癌检查方式^[33]^。针对术前CT表现为纯磨玻璃影IA的术后病理亚型分析结果显示，其大多为预后较好的三种亚型——贴壁生长成分（47.4%-66.7%）、腺泡（13.3%-42.1%）以及乳头状（7.3%-10%）^[34,35]^。然而，一项meta分析^[36]^发现，仅凭CT特征无法区分磨玻璃结节为IA或是浸润前病变。Li等^[37]^发现双能CT中的肿瘤大小、空气支气管征、K40e65 keV和CT平扫有效原子数与水浓度是预测SPA的最有效变量，其受试者工作特征（receiver operating characteristic curve, ROC）曲线下面积为0.906。因此，虽然目前还无法通过CT特征预测肺结节的病理亚型，但未来仍有望通过双能CT等方法进行IA的病理亚型预测。

正电子发射断层扫描/CT（positron emission tomography/CT, PET/CT）是临床评估肿瘤的淋巴结转移、远处转移以及侵袭性的标准。最大标准化摄取值（maximal standardized uptake value, SUVmax）代表肿瘤糖酵解代谢，高SUVmax提示高侵袭性。有meta分析^[38,39]^显示高SUVmax的肿瘤预后较差。Fujikawa等^[24]^的研究中，肿瘤SUVmax中位数与肿瘤成分中各预后级别的构成呈现出一致性，提示高预后级别INMA糖酵解代谢更加活跃。Yang等^[40]^发现，不同病理亚型IA的SUVmax不同，其中位数分别为：MPA 9.93（2.74, 16.72）、SPA 8.4（4.53, 11.44）、PPA 6.55（2.52, 8.21）、APA 5.78（2.44, 9.08）、LPA 2.2（1.81, 3.16）。Bu等^[41]^也观察到IA的5种病理亚型中MPA的SUVmax最高，LPA的SUVmax最低。上述研究显示，SUVmax对不同病理亚型INMA的鉴别具有一定价值，虽然目前已阐明高预后级别的病理亚型SUVmax值较高，但仍无法通过SUVmax推测肿瘤的病理亚型，希望未来有更多这方面的研究数据来帮助医师准确预测。

影像组学通过自动化或半自动化的大通量提取来自放射成像的可量化信息，可与基因组数据结合，用以识别肿瘤遗传表型、预测疾病的潜在预后和对治疗的反应^[42⇓-44]^。影像组学已广泛应用于肿瘤学研究，影像组学对鉴别良恶性肺结节以及肺腺癌病理亚型方面的效果已被证实^[45,46]^。Chen等^[47]^证明了影像组学特征在预测高级别MPA和SPA病理亚型成分方面具有高敏感性和中等特异性。利用影像组学对CT图像的特征进行早期识别，能识别出高风险患者，辅助临床医生制定治疗方案。

深度学习是在高抽象水平上自动提取复杂数据特征的一种新兴的模式分析方法^[48]^。与影像组学方法相比，深度学习模型具有明显的优势，因其支持在不需人工干预的情况下以增量方式进行特征提取，有望应用于各种成像模式的结果分析^[49]^。已有学者^[50,51]^通过纳入数据集来建立深度学习模型，该模型可较准确地预测微乳头状和实体亚型等高预后级别病理亚型成分。虽然目前尚无针对其他病理亚型的研究，但深度学习仍是具有潜力的病理亚型预测工具。

通过影像学来开发非侵入性的肺癌亚型分类方法可以帮助医生更好地做出治疗决策，还可以为无法获得足够组织进行病理学检查的患者提供影像学诊断方法。

## 4 病理亚型与淋巴结转移

淋巴结转移是癌细胞从原发肿瘤向远处器官转移的重要途径，同时也是影响肺癌治疗和预后的重要因素，淋巴结受累与预后显著相关^[52]^。含高侵袭性成分的IA更容易发生淋巴结转移^[53]^。根据肺腺癌IASLC/ATS/ERS国际多学科分类，各病理亚型发生淋巴结转移的频率有所差异，微乳头及实体为主的亚型更具侵袭性，更易发生淋巴结转移^[54]^。一项国内回顾性研究^[55]^纳入了2,268例经根治性手术切除的肺腺癌患者，部分病例中的病理亚型组成为混合成分，该研究发现肿瘤中存在微乳头型和实体型成分的淋巴结转移患者分别有1,068例和1,080例，远高于含有腺泡型和乳头型成分的429例和411例，证实了SPA和MPA的淋巴结转移率明显高于其他亚型。Yuan等^[56]^进行的一项大规模队列研究分析表明，微乳头状（OR=3.51, 95%CI: 2.09-5.89）和实体成分（OR=2.28, 95%CI: 1.42-3.64）的存在是淋巴结升期的独立预测因素，能显著增加术后淋巴结升期的风险，并与不良预后独立相关。Zhao等^[57]^研究认为IA表现出高度的异质性，微乳头成分≥5%的患者较无微乳头成分的患者有更高的淋巴结转移及肿瘤复发风险。Lin等^[58]^研究表明，贴壁生长为主型IA[3.2%（1/31）]不易发生淋巴结转移，伴淋巴结转移者也多为N1淋巴结转移，但实体[100%（4/4）]和微乳头状成分[11.8%（2/17）]的淋巴结转移率明显高于其他病理亚型，淋巴结分期也较高。Chang等^[59]^也认为实体及微乳状成分[46.88%（45/96）]的存在会发生较高的淋巴结转移率。隐匿性淋巴结转移是指常规病理学检查未能发现的淋巴结微小转移病灶^[60]^。Hung等^[61]^发现肺腺癌中微乳头状及实体成分的存在与隐匿的N2淋巴结转移显著相关。

综上所述，微乳头状及实体成分的存在可能是淋巴结转移的预测指标，可作为临床医师选择手术及术后治疗方式的依据。但目前尚无任何研究表明各病理亚型与肿瘤侵袭性及远处血行转移相关。

## 5 病理亚型与手术及术后化疗

手术切除是治疗早期肺癌最有效的方式^[62]^。肺叶切除术结合系统性淋巴结清扫是金标准^[63]^。随着影像技术的发展，对于部分I期肺腺癌，亚肺叶切除（肺段切除和楔形切除）可取得与肺叶切除相似的预后，同时又能保留更多正常的有功能的肺组织^[64,65]^。日本临床肿瘤学会（Japan Clinical Oncology Group, JCOG）0802的研究^[66]^结果显示，亚肺叶切除已经获得广泛应用，但对于其远期生存率仍然有争议^[67]^。病理亚型是在I期肺腺癌切除方式选择上除肿瘤大小之外的一个重要的指征^[68]^。Cox等^[69]^分析了1,991例cT1-2N0M0的LPA患者，与亚肺叶切除术相比，肺叶切除术并未显著改善患者生存率（HR=0.99, 95%CI: 0.77-1.27）。结果显示在I期LPA中行亚肺叶切除是可行的，但要在充分进行病理淋巴结的评估下谨慎选择亚肺叶切除术。Choi等^[13]^调查了具有极少实体或微乳头状成分且接受手术治疗的506例肺腺癌患者的临床特征及预后，证明了即使在IA期肺腺癌中，极少的实体或微乳头状成分存在也与侵袭性临床病理特征和不良预后显著相关，Zhao等^[54]^和Yanagawa等^[70]^的研究也得出了类似的结果。Nitadori团队^[71,72]^通过研究接受肺叶和亚肺叶切除的I期浸润型肺腺癌（≤2 cm）患者术后复发的情况，认为在微乳头成分≥5%的患者中，亚肺叶切除组的5年复发率为34.2%，明显高于肺叶切除组的复发率（19.1%），提示微乳头成分≥5%是IA（≤2 cm）患者接受亚肺叶切除术的术后复发独立危险因素。Su等^[10]^认为对于影像学提示为部分实性结节且肿瘤微乳头成分≥5%的患者，肺叶切除术有比亚肺叶切除术更好的总生存率（overall survival, OS）和无复发生存率（recurrence-free survival, RFS）。综上，我们建议针对微乳头成分或实体成分≥5%的I期肺腺癌患者应采用肺叶切除术，而其他病理亚型的患者若亚肺叶切除可行，均可采用亚肺叶切除术。

对于早期肺腺癌术后辅助治疗，美国国立综合癌症网络（National Comprehensive Cancer Network, NCCN）指南不建议IA期的患者行术后辅助治疗^[73]^。对于可手术切除的IIA期-IIIB期的非小细胞肺癌（non-small cell lung cancer, NSCLC）患者，目前术后含铂类药物的化疗已经是常规的术后辅助治疗，但对于IB期患者是否行术后辅助治疗存在争议。国外学者^[74]^对725例肺腺癌患者进行术后辅助化疗的疗效研究，发现术后辅助化疗可提高IB期SPA和MPA患者的无病生存率（disease-free survival, DFS）（HR=0.60, 95%CI: 0.44-0.82），Luo等^[75]^和Qian等^[76]^的研究也得出了相同的结论，但该研究未涉及伴有少量微乳头状和实体成分的腺癌患者能否从辅助化疗中获益。Wang等^[77]^通过大规模的meta分析证明微乳头成分存在有着更差的DFS（HR=1.62, 95%CI: 1.20-2.21）和OS（HR=1.53, 95%CI: 1.19-1.96），是IA患者的不良预后因素，伴有少量微乳头成分的患者在R0切除后能受益于辅助化疗。虽然SPA和MPA的IB期患者可从术后化疗中获益，但目前国内外指南均未建议上述两种病理亚型患者进行术后化疗，也许未来仍需大样本、多中心、前瞻性的研究对本问题进行解答。

## 6 病理亚型与驱动基因突变

驱动基因是在多种癌症中发挥关键功能的基因类型，识别肿瘤发生和发展过程中驱动基因的突变是至关重要的^[78]^。在肺癌中，驱动基因突变最常出现于腺癌中，常见的基因突变类型有表皮生长因子受体（epidermal growth factor receptor, EGFR）、间变性淋巴瘤激酶（anaplastic lymphoma kinase, ALK）、Kirsten大鼠肉瘤病毒癌基因同源物（Kirsten rat sarcoma viral oncogene homolog, KRAS）^[79]^。不同病理亚型INMA的驱动基因突变情况不同，在过去的十多年里，多个团队研究了病理亚型与驱动基因突变状态之间的关系。

Shim等^[80]^的研究发现超过50%手术切除的IA中存在EGFR突变，EGFR突变与微乳头成分和贴壁生长成分比例存在显著关联。也有研究报道，EGFR突变在LPA（44%-77%）^[81⇓⇓⇓-85]^、APA（44%）^[86]^、PPA（56%）^[87]^和MPA（30%-63.9%）中更为频繁^[78,88,89]^，整体来看SPA（2.6%-28%）突变率较低。de Melo等^[90]^发现KRAS突变与APA显著相关。然而，Dong等^[91]^发现KRAS突变更常见于SPA中。不难发现，上述研究中EGFR/KRAS突变和病理亚型之间的关系并不统一。为解决这些争议，Jiang等^[92]^通过数据库纳入了9,022例基因突变患者的资料进行了大型的meta分析，结果表明EGFR突变在LPA（OR=1.76, 95%CI: 1.38-2.24）中更为常见，而在IMA（OR=0.10, 95%CI: 0.06-0.14）和SPA（OR=0.28, 95%CI: 0.23-0.34）中很少被发现，该研究并未发现EGFR突变与APA、PPA和MPA之间有任何显著关联。KRAS突变在IMA（OR=7.01, 95%CI: 5.11-9.62）中更常见，而在LPA（OR=0.58, 95%CI: 0.45-0.75）和APA（OR=0.65, 95%CI: 0.55-0.78）中较少被发现。该研究还发现亚洲人群和非亚洲人群的EGFR突变与病理亚型的关系呈现出一致性。Fujikawa等^[24]^证实了根据新的IASLC分级系统，EGFR突变在1级和2级肿瘤中更为频繁（71.6%）；KRAS突变在3级中更占优势（65.0%）。整体来说，EGFR突变在中高分化的病理亚型中更常见，KRAS突变在低分化的病理亚型中更频繁。这可能是SPA和IMA预后更差的原因之一。

上海市胸科医院对手术切除的2,299例肺腺癌患者进行了一项ALK重排与肺腺癌病理亚型之间关系的探究，结果显示2,299例肿瘤中有93例（4.0%）发现ALK重排。ALK重排频率分别为：SPA 10.3%（20/195）、MPA 7.6%（13/170）、APA 2.8%（29/1,035）、PPA 2.0%（11/539）、LPA 0.9%（1/114），ALK重排在SPA和MPA中显著高于其他亚型。Deng等^[21]^也发现ALK重排在高级别肿瘤中更为普遍。ALK重排有着独特的临床病理特征，Dong等^[91]^学者发现25%（13/52）伴黏蛋白产物的SPA伴有ALK重排，存在印戒细胞和筛状结构的SPA也与ALK重排相关。

目前不同研究所显示的驱动基因与病理亚型关系不一致的原因尚不明确，未来还需要更多的研究来探讨不同驱动基因与病理亚型的关系，今后有望通过肺癌病理亚型指导患者的基因检测选择。

## 7 病理亚型与免疫检查点抑制剂

近年，对于不可手术切除的中晚期NSCLC，免疫检查点抑制剂的出现使其治疗方案发生了巨大的变化。程序性细胞死亡配体1（programmed cell death ligand 1, PD-L1）的肿瘤细胞阳性比例分数（tumor proportion score, TPS）是预测免疫治疗有效性最常用的生物标志物^[93]^。Cruz-Rico等^[94]^研究发现PD-L1阳性（TPS≥1%）和高预后级别INMA显著相关，SPA的肿瘤样本TPS≥1%的比例（68.7%）高于其他病理亚型。在Miyazawa等^[95]^的研究中，PD-L1阳性（TPS≥1%）在INMA中的表达频率分别为SPA（73.7%）、APA（49.2%）、PPA（43.3%）、MPA（40.0%），PD-L1的TPS≥50%在SPA中的表达频率最高（53.6%）。INMA的实体型成分可能代表更高的PD-L1的TPS，并提示更好的免疫治疗疗效。另一些研究^[88,96,97]^表明，尽管实体或微乳头亚型都有很强的侵袭性，但它们在EGFR突变和PD-L1表达方面均存在显著差异，这可能与这两种病理亚型具有不同的生物学背景有关，因此MPA和SPA可能通过不同机制导致患者预后不良。目前鲜有关于PD-L1表达与病理亚型的大样本研究，无法得出较为客观的病理亚型与PD-L1表达的关系，同时对于不同病理亚型INMA患者的免疫治疗疗效也需进一步探究。

## 8 展望

2021年WHO第5版胸部肿瘤分类的公布对INMA提出明确的亚型分类，并推荐使用新的IASLC分级系统，为未来的研究提供了更加标准的分类规范。现阶段关于INMA病理亚型的研究已经取得了一些成果，但仍有较大的研究空间，如通过影像组学和人工智能（artificial intelligence, AI）等无创方法来预测病理亚型；对于早期肺腺癌，应该如何结合病理亚型来选择肺叶或亚肺叶切除的手术方案；分子靶向治疗和免疫治疗对不同病理亚型的治疗获益。未来仍需要更多关于不同病理亚型的INMA特征的研究来解决这些问题。
